# Herbal Medicine AC591 Prevents Oxaliplatin-Induced Peripheral Neuropathy in Animal Model and Cancer Patients

**DOI:** 10.3389/fphar.2017.00344

**Published:** 2017-06-07

**Authors:** Xiaolan Cheng, Jiege Huo, Dawei Wang, Xueting Cai, Xiaoyan Sun, Wuguang Lu, Yang Yang, Chunping Hu, Xiaoning Wang, Peng Cao

**Affiliations:** ^1^Affiliated Hospital of Integrated Traditional Chinese and Western Medicine, Nanjing University of Chinese MedicineNanjing, China; ^2^Laboratory of Cellular and Molecular Biology, Jiangsu Province Academy of Traditional Chinese MedicineNanjing, China

**Keywords:** oxaliplatin, herbal medicine, AC591, peripheral neuropathy, antitumor activity

## Abstract

Oxaliplatin is clinically compelling because of severe peripheral neuropathy. The side effect can result in dosage reductions or even cessation of chemotherapy, and no effective treatments are available. AC591 is a standardized extract of Huangqi Guizhi Wuwu decoction, an herbal formula recorded in “Synopsis of the Golden Chamber” for improving limb numbness and pain. In this study, we investigated whether AC591 could protect against oxaliplatin-induced peripheral neuropathy. To clarify it, a rat model of oxaliplatin-induced peripheral neuropathy was established, and neuroprotective effect of AC591 was studied. Our results showed that pretreatment with AC591 reduced oxaliplatin-induced cold hyperalgesia, mechanical allodynia as well as morphological damage of dorsal root ganglion. Microarray analysis indicated the neuroprotective action of AC591 depended on the modulation of multiple molecular targets and pathways involved in the downregulation of inflammation and immune response. Moreover, AC591 enhanced the antitumor activity of oxaliplatin to some extent in Balb/c mice bearing CT-26 carcinoma cells. The efficacy of AC591 is also investigated in 72 colorectal cancer patients. After four cycles of treatment, the percentage of grades 1–2 neurotoxicity in AC591-treated group (*n* = 36) was 25%, whereas in the control group the incidence was 55.55% (*P* < 0.01) (*n* = 36). No significant differences in the tumor response rate between the two groups were found. These evidences suggested that AC591 can prevent oxaliplatin-induced neuropathy without reducing its antitumor activity, and may be a promising adjuvant to alleviate sensory symptoms in clinical practice.

## Introduction

Oxaliplatin is a widely used chemotherapeutic agent for cancers including colorectal, lung, ovarian, and pancreatic (André et al., 2004). However, about 85–95% of cancer patients receiving oxaliplatin suffer from serious peripheral neuropathy, characterized by stocking-and-glove distribution sensory loss, paresthesia, dysesthesia, and pain (Coriat et al., 2014; Wolf et al., 2008). Oxaliplatin-induced neurotoxicity is classified into acute and chronic forms. The acute neuropathy appears within hours of oxaliplatin infusion, accompanied by transient and reversible symptoms including hyperpathic pain triggered by cold (Wilson et al., 2002). Chronic neurological syndrome develops after a cumulative dose of oxaliplatin (540 mg/m^2^ over four cycles or more of therapy) and consist of sensory impairment of peripheral nerves with distally pronounced dysesthesias and paresthesias of the extremities (Argyriou et al., 2012; Pachman et al., 2015). Although many studies have attempted to describe the pathophysiology of oxaliplatin-induced neuropathy, the underlying mechanisms remain poorly understood. Recently, several hypotheses have been proposed to explain oxaliplatin neurotoxicity, including mitochondrial dysfunction, increased the content of oxidative substances, altered function of different ion channels, dysregulation of Ca^2+^ homeostasis, membrane drug transporters, and central glia in the dorsal root ganglion (Mantyh, 2006; Pabbidi et al., 2008; Di Cesare Mannelli et al., 2012; Jaggi and Singh, 2012; Wang et al., 2012; Sprowl et al., 2013). Nonetheless, unified mechanisms that may confirm the clinical and experimental results have hitherto not been advanced.

Oxaliplatin-induced peripheral neuropathy may result in dosage reductions and delays, and in some cases cessation of oxaliplatin injection (Beijers et al., 2014). Several types of analgesics, anticonvulsants, and antioxidants have been tested for preventing neurotoxicity induced by oxaliplatin (Ta et al., 2010; Di Cesare Mannelli et al., 2015; Li et al., 2015; Liu and Huang, 2015; Martyn and Petito, 2015). However, most of these drugs either showed little efficacy in double-blinded, placebo-controlled trials, or cause unexpected side-effects (Gamelin et al., 2008; Gutiérrez-Gutiérrez et al., 2010; Loprinzi et al., 2014). Currently, safe and efficient pharmacotherapeutics for oxaliplatin-induced neuropathy are still unavailable. Identifying useful neuropathy-reducing agents has been considered a significant unmet medical prerequisite.

AC591 is derived from the classical formulation of Huangqi Guizhi Wuwu decoction (HGWD), which was first described in “Synopsis of the Golden Chamber” in the beginning of the third century for treating numbness, vibration sensation, cold sensation, and limb ache (Zhang, 2013). HGWD is composed of 18 g Astragali Radix [AR, the dry roots of *Astragalus membranaceus* (Fisch.) Bge. var. *mongholicus* (Bge.) Hsiao or *A. membranaceus* (Fisch.) Bge., Leguminosae], 9 g Cinnamomi Ramulus (CR, the dry tender branches of *Cinnamomum cassia* Presl., Lauraceae), 9 g Paeonia Radix Alba (PRA, the dry roots of *Paeonia lactiflora* Pall., Ranunculaceae), 9 g Jujubae Fructus (JF, the dry ripe fruits of *Ziziphus jujuba* Mill., Rhamnaceae), and 9 g Zingiberis Rhizoma (ZR, the dry rhizome of *Zingiber officinale* Rosc., Zingiberaceae). These crude drugs have been widely used for their multifunctional activities such as antioxidation, anti-inflammation, and neuroprotection (Fu et al., 2014). Recently, HGWD has been shown to ameliorate decreased nerve conduction velocity and a variety of subjective symptoms associated with diabetic peripheral neuropathy in clinical studies (Tong and Hou, 2006). In one case report, HGWD was recorded to show an antiallodynic effect on neuropathic pain caused by oxaliplatin, thus allowing the continuation of the suspended chemotherapy (Tatsumi et al., 2009). Despite the potential of HGWD as a neuroprotective agent, no experimental study has been conducted to determine its effect in animal models of oxaliplatin-induced neuropathy. Moreover, the impact of HGWD on the antitumor activity of oxaliplatin remains unknown. In this work, we aimed to clarify whether AC591, a standardized extract of HGWD, is efficient in reducing the oxaliplatin-related peripheral neuropathy and anti-tumor activity in rodents and clinical trials.

## Materials and Methods

### Materials and Reagents

AR, CR, PRA, JF, and ZR were purchased from Jiangsu Hospital of Integrated Traditional Chinese and Western Medicine. All the crude drugs were morphologically authenticated according to Chinese Pharmacopoeia (2010 Edition). The voucher specimens were deposited at Laboratory of Cellular and Molecular Biology, Jiangsu Province Academy of Traditional Chinese Medicine, Nanjing, China. Reference substances including gallic acid, paeoniflorin, calycosin-7-*O*-β-D-glucoside, rutin, coumarin, ononin, cinnamic acid, formononetin, astragaloside IV, 6-gingerol and 6-shogaol were purchased from Must Biological Technology Co. Ltd (Chengdu, China). The purity of each reference compound was over 98% by HPLC.

HPLC-grade acetonitrile and formic acid were purchased from ROE Scientific Inc. (United States). Deionized water was purified using a Milli-Q water purification system from Millipore (Bedford, MA, United States).

### AC591 Preparation and Quality Control

To prepare AC591 samples, AR, CR, PRA, JF, and ZR were mixed at a ratio of 2:1:1:1:1 and refluxed for 2 h with 10 volumes of water after maceration for 30 min. The water extract was filtered, and the residue refluxed again with 8 volumes of water for 2 h. The combined filtrates were desiccated to produce the AC591 extract at a concentration of 20 g crude drug/mL, and stored at -80°C. The extracts were filtered through a 0.2 μm PTFE syringe filter.

Qualitative analysis was performed by HPLC-MS/MS system consisting of Waters 2695 HPLC instrument and Quattro Premier XE MircoMass triple quadrupole tandem mass spectrometer (Waters Co., Milford, MA, United States). Chromatographic separation was carried out at 35°C on a Waters MS C_18_ column (250 mm × 4.6 mm, 5.0 μm) using the following gradient profile: 5–15% B (0–0.15 min), 15–20% B (15–35 min), 20–40% B (35–65 min), 40–60% B (65–75 min), 60–100% B (75–90 min). The mobile phase was composed of 0.1% acetic acid (A) and 100% acetonitrile (B). The injection volume was 2 μL. Quantification analysis was carried out using HPLC-DAD method. The concentrations of major constituents were determined by an external standard curve.

### Animals

Male Wistar rats (200–250 g) for oxaliplatin-induced peripheral neuropathy model and 6-week-old male BALB/c mice (21–23 g) for *in vivo* tumor growth model were feeded in Experimental Animal Center, Jiangsu Academy of Traditional Chinese Medicine (Nanjing, China). Animals had free access to food and water in their home cages. All experimental procedures were approved by the Animal Care and Use Committee at Jiangsu Academy of Traditional Chinese Medicine. Efforts were made to minimize the number of animals used and their suffering during the study.

### Experimental Design and Protocol

Fifty male wistar rats were randomized into five experimental groups (*n* = 10): vehicle, oxaliplatin, oxaliplatin +AC591 (5 g/kg), oxaliplatin+AC591 (10 g/kg), and oxaliplatin+AC591 (20 g/kg). Oxaliplatin was obtained from Jiangsu Hengrui Medicine Co., Ltd (Jiangsu, China) and solubilized in 5% glucose solution. In vehicle and oxaliplatin groups, 5% glucose solution or oxaliplatin (4 mg/kg) was, respectively, injected intraperitoneally (i.p.) twice a week for 4 weeks (on days 1, 2, 8, 9, 15, 16, 22, and 23). In AC591-treated groups, in addition to oxaliplatin injection (4 mg/kg, i.p., twice a week), AC591 extract was administered orally daily for 4 weeks. The dosage of oxaliplatin was chosen based on a previous report (Ushio et al., 2012). Concurrently, the body weight of the rats was measured, and behavioral tests were performed blindly with respect to drug administration. At the end of the experiment, dorsal root ganglion (DRG) and sciatic nerves were harvested from all rats.

### Von Frey Test for Mechanical Allodynia

The mechanical allodynia induced by oxaliplatin was assessed by Von Frey hair test before the first administration of drugs (on day 0) and on days 7, 14, 22, and 25. The rats were placed in a clear plastic box (20 cm × 17 cm × 13 cm) with a wire mesh floor and allowed acclimatization for 30 min prior to testing. Von Frey filaments (Stoelting, Wood Dale, IL, United States) ranging from 1 to 26 g bending force were applied to stimulate the mid-plantar skin of each hind paw at 3–4 s intervals. The minimal pressure level [in gram (g)] that initiated a withdrawal response was recorded. A positive response was noted if the tested paw was withdrawn sharply during 5 of the 10 trials.

### Cold Plate Test and Acetone Test for Cold Hyperalgesia

Cold hyperalgesia was assessed at 4°C using a cold plate. Cold hyperalgesia was measured as the number of foot withdrawal responses (lifting, shaking, or licking) in 1 min after the application of cold stimuli to the plantar surfaces of both the paws. The testing was repeated 3 times with an interval of approximately 5 min between each test. The cold hyperalgesia was also assessed by acetone test. Rats were placed in a clear plastic box (20 cm × 17 cm × 13 cm) and allowed to habituate for 30 min prior to testing. Acetone (50 μL) was sprayed onto the plantar skin of each hind paw, and the number of withdrawal response was counted for 40 s from the start of the acetone spray. The results were reported as the mean of 3 readings.

### Hot Plate Test for Thermal Sensing

The noxious heat thresholds for heat hyperalgesia were measured using an increasing-temperature hot plate (IITC Life Science). After habituation, the rats were placed onto the plate, which was then heated from a starting temperature of approximately 30°C at a rate of 12°C/min until the animal showed nocifensive behavior. The corresponding plate temperature was considered as the noxious heat threshold. All results were reported as the mean of 2 readings from both the hind paws.

### Histopathological Analysis

On day 28, sciatic nerves and DRG were harvested from rats anesthetized with sodium pentobarbital. The nerves were fixed in 10% paraformaldehyde, washed with saline, dehydrated by incubation in different concentrations of ethanol, and rinsed with xylene. Next, they were paraffin embedded, and the tissue was cut to a thickness of 5 μm, followed by hematoxylin and eosin (H&E) staining. Pathological changes were evaluated under a light microscope (BX51; Olympus Corp., Tokyo, Japan).

### Gene Expression Microarray Analysis

DRG tissues of rats from the vehicle, oxaliplatin, oxaliplatin+AC591 (20 g/kg) groups were subjected to total RNA extraction and microarray hybridization. The Rat 12 × 135K Gene Expression Array manufactured by Roche NimbleGen was used. Double-strand cDNA (ds-cDNA) was synthesized from total RNA using an Invitrogen SuperScript ds-cDNA synthesis kit in the presence of 100 pmol oligo dT primers. ds-cDNA was purified and labeled in accordance with the NimbleGen Gene Expression Analysis protocol (NimbleGen Systems, Inc., United States). The purified cDNA was quantified by a NanoDrop ND-1000. For Cy3 labeling of cDNA, the NimbleGen One-Color DNA labeling kit was used according to the manufacturer’s guidelines (NimbleGen Systems, Inc., Madison, WI, United States). Microarrays were hybridized at 42°C for 16 to 20 h with 4 μg of Cy3 labeled ds-cDNA in NimbleGen hybridization buffer/hybridization component A in a hybridization chamber (Hybridization System – NimbleGen Systems, Inc., Madison, WI, United States). Following hybridization, the microarrays were washed with the NimbleGen Wash Buffer Kit. The slides were scanned in an ozone-free environment using the Axon GenePix 4000B microarray scanner (Molecular Devices Corporation) piloted by GenePix Pro 6.0 software (Axon).

The scanned images (TIFF format) were then imported into NimbleScan software (version 2.5) for grid alignment and data analysis. The expression data were normalized through quantile normalization and the Robust Multichip Average (RMA) algorithm included in the NimbleScan software. The Probe level (^∗^_norm_RMA.pair) files and Gene level (^∗^_RMA.calls) files were generated after normalization. All gene level files were imported into Agilent GeneSpring GX software (version 12.0) for further analysis. Differentially expressed genes with statistical significance were identified through Volcano Plot filtering. Hierarchical clustering was performed with the R software (version 2.15). GO and pathway analyses were performed using the standard enrichment computation method.

### Tumor Cytotoxicity Assay

CT26 (2 × 10^6^) cells were injected subcutaneously into the back of 6-week-old male BALB/c mice (21–23 g). When the tumors had grown to 200–300 mm^3^ size (day 10), mice were randomly assigned to five groups (*n* = 8 each). Vehicle group was treated by intragastrical saline (0.2 mL) for 2 weeks, and oxaliplatin group was processed by intraperitoneal oxaliplatin (10 mg/kg/week, 2 weeks, i.p.). The groups treated with oxaliplatin and AC591 were administrated oxaliplatin (10 mg/kg/week), and AC591 (7, 14, and 28 g/kg, respectively, i.g. daily for 14 days). Tumor size was measured with a caliper rule every 3 days, and the volume was calculated as follows: TV (mm^3^) = (L × W^2^)/2, wherein *L* is the longest and *W* the shortest radius of the tumor (mm). At the end of the treatment period, the mice were sacrificed, and tumors were weighed.

### Clinical Study

The clinical trial was reviewed and approved by the ethics committee of Jiangsu Hospital of Integrated Traditional Chinese and Western Medicine (2013LW029). This trial was registered at Chinese Clinical Trials Registry (ChiCTR-TRC-14004765) on 6th June 2014, and conformed to the CONSORT flowchart (**Figure 6**) and checklist. Written consent was obtained from each patient.

#### Eligibility Criteria

Patients with colon or rectum cancer scheduled to undergo FOLFOX chemotherapy were enrolled in this study from the oncology department of Jiangsu Hospital of Integrated Traditional Chinese and Western Medicine. All eligible patients were required to have a satisfactory performance status (ECOG 0-2), life expectancy over 6 months, adequate hematopoietic function in order to undergo chemotherapy, normal hepatic function test (aspartate aminotransaminase and alanine aminotransferase ≤1.5 times the normal upper limit), and renal function test (serum total bilirubin and creatinine each <1.5 mg/dL). Exclusion criteria comprised of hypercalcemia, history of alcoholic intoxication, diabetes, central nervous system metastasis, preexisting neuropathy, or prior use of antidepressant, anticonvulsant, or other neuropathic pain medications. Patients who were pregnant or nursing were also excluded from the trial.

#### Treatment

A total of 82 cancer patients were assessed for eligibility in this trial. Out of them, 10 patients decided against participation in the study. Finally, 72 patients were randomly allocated to the study treatment in the AC591 and Non-AC591 groups. Selection for AC591-treated group was determined by a computer-generated randomization list with the help of a professional statistician in a 1:1 ratio, and the random number table was sealed in a special envelope. The drug administrators, patients and researchers were all unaware of the blind design. The evaluators and the statisticians were not involved in the trial. The patient characteristics such as age, sex, activity level, the location of primary tumor, cancer stage, and metastasis were analyzed.

Patients in AC591 group were scheduled to receive at least four cycles (2 months) of oxaliplatin-based FOLFOX chemotherapy plus AC591. The AC591 includes AR 18g, CR 9g, PRA 9g, JF 9g, and ZRR 9g in one unit. AC591 and placebo drug were provided and quality controlled by the hospital pharmacy. All patients in AC591-treated group were asked to take AC591 twice per day (in the morning and in the evening) for a total of 54 g crude drug/day. The appearance, smell and taste of the placebo drug were disguised to be identical to the treatment drug. All the patients underwent follow-up office visits every 2 weeks for 2 months to monitor the incidence and severity of various side effects. Participants recruited into the trial would be withdrawn if they were lost to follow-up, refused enemas, dropped out, or had serious adverse effects.

#### Study Endpoints

The primary study endpoints included oxaliplatin-induced neurotoxicity and tumor response. The severity of peripheral neuropathy was evaluated and graded into five categories according to the oxaliplatin-specific Levi’s scale as follows: grade 0, asymptomatic; grade 1, mild sensory alteration or paresthesia (not requiring intervention); grade 2, sensory alteration or paresthesia that moderately interferes with instrumental activities of daily living (ADLs); grade 3, sensory alteration or paresthesia that severely interferes with basic ADLs; grade 4, disabling neuropathy. The clinical and tumor responses (complete response, partial response, progressive disease, or stable disease) were assessed at the end of the clinical trial by CT scans according to World Health Organization criteria.

### Statistics

For animal experiments, the values are represented as mean ± SD. Statistical significance between the groups was analyzed by Student *t*-test. The differences were considered statistically significance at *P* < 0.05. For the human study, the rates of neurotoxicity were calculated for each grade and compared between trial and control group. The difference in the grades of neurotoxicity and tumor response between two groups was compared using Chi-square test and *P* < 0.05 defined as statistically significant.

## Results

### Quality Control Analysis of AC591

**FIGURE 1:**
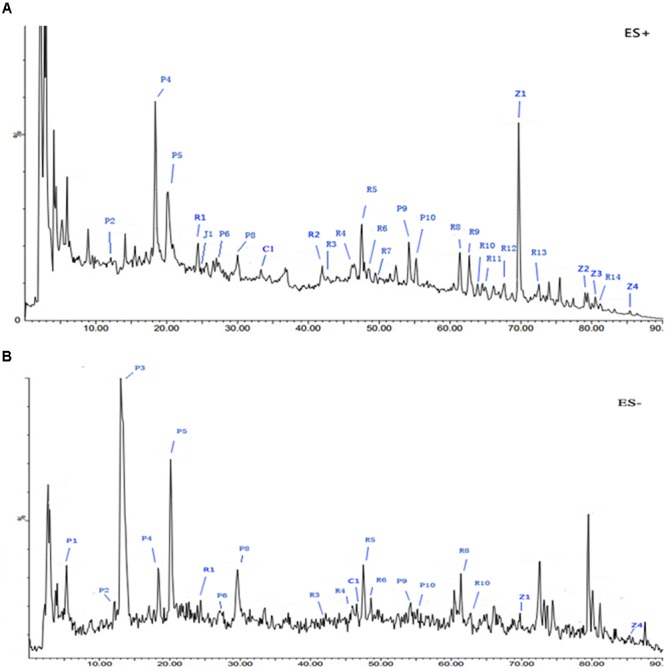
Representative base peak intensity (BPI) chromatograms of AC591 analyzed by LC-MS in positive **(A)** and negative **(B)** mode.

The spectral profile of AC591 is presented in **Figure 1**, and the enumerated peaks from the chemical fingerprint are listed in Supplementary Table 1. A total of 36 individual phytochemical peaks were used to define the overall chemical fingerprint pattern, constituting >85% of the total ion current. Among the identified compounds, 25 were characterized using markers, exact mass comparison, and MS/MS fragmentation. Moreover, most of the molecular ions in the fingerprint arise from AR (41.67%) followed by PRA (27.78%), CR (16.67%), ZR (11.11%), and JF (2.78%). The 36 components, uniquely identified to 1 out of the 5 main herbs, could result in a signature of the composition, extraction method, and the herbal ratios. To standardize AC591, up to six marker compounds for main herbal ingredient were quantitatively analyzed: paeoniflorin (PRA), gallic acid (PRA), albiflorin (PRA), ononin (AR), cinnamic acid (CR), and 6-ginger (ZR). Using linear regression analysis on the commercial markers as standards, the amount of AC591 extract phytochemical (mg/g) was determined. The reproducibility, recovery, intra-day and inter-day analyses, and contents of the compounds are summarized in **Table 1**. The combination of chemical fingerprinting and quantitation of a subset of phytochemical markers provide a global quality control of AC591.

**Table 1:** Calibration curves, limit of detection (LOD), limit of quantification (LOQ), accuracy, recovery, and contents of six analytes in AC591.

Analytes	Calibration curve	LODs (μg/mL)	LOQs (μg/mL)	Precision (RSD%)		Average recovery %	Contents (μg/g)
				Intra-day (*n* = 6)	Inter-day (*n* = 9)		
Gallic acid	*y* = 639.1x-653.5	0.3	1.17	2.04	1.21	98.36	417.40
Paeoniflorin	*y* = 84.5x-25.5	0.21	0.85	1.39	2.34	104.17	3290.00
Albiflorin	*y* = 204.0x-60.6	0.29	0.56	2.00	3.14	98.62	372.21
Ononin	*y* = 128.7x-24.4	0.10	0.37	1.50	2.83	97.50	160.02
Cinnamic acid	*y* = 1189.5x-278.7	0.10	0.40	1.16	2.21	100.39	126.36
6-Gingerol	*y* = 196.9x-51.2	0.24	0.47	2.86	2.48	99.36	106.79

### AC591 Treatment Did Not Attenuate Oxaliplatin-Induced Body Weight Loss

**FIGURE 2:**
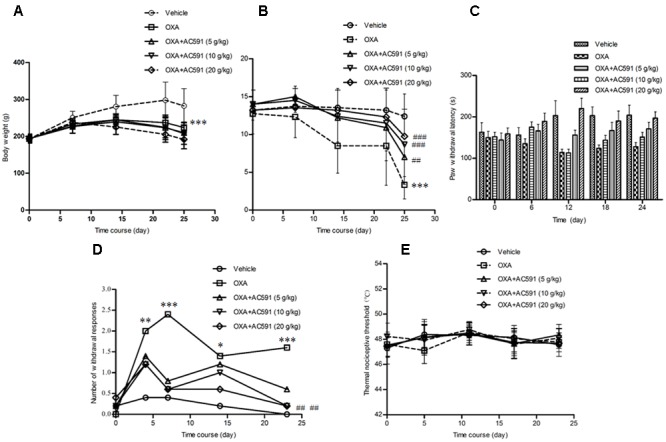
Effects of repeated administration of AC591 on oxaliplatin-induced body reduction **(A)**, mechanical allodynia **(B)**, cold hyperalgesia (**C**, cold plate test; **D**, acetone test), and thermal sensing **(E)** in rats. Each value represents the mean of 10 rats per group, performed in two different experimental sets. ^∗^*P* < 0.05, ^∗∗^*P* < 0.01, ^∗∗∗^*P* < 0.001 vs. vehicle; ^#^*P* < 0.05, ^##^*P* < 0.01, ^###^*P* < 0.001 vs. oxaliplatin.

The effects of AC591 on body weight loss in the oxaliplatin-induced neuropathy model was evaluated. The results are shown in **Figure 2A**. It was found that the rats started to lose weight after the first injection of oxaliplatin, which reached statistical significance after the fourth dosage as compared to the vehicle-treated rats. None of the rats in the vehicle control group exhibited mortality during the experiment. While in the oxaliplatin-treated group (4 mg/kg), two rats perished after eight injections and eight other rats turned slim at the end of the treatment. Unexpectedly, AC591 (5, 10, and 20 g/kg) treatment did not attenuate oxaliplatin-induced body weight loss.

### AC591 Treatment Prevented Oxaliplatin-Induced Mechanical Allodynia

Oxaliplatin was reported to lower the rat’s threshold to mechanical stimuli which do not normally invoke pain (Ushio et al., 2012). Using the Von Frey apparatus, the withdrawal threshold to the non-noxious mechanical stimulus was estimated. As shown in **Figure 2B**, oxaliplatin caused a lowering of response threshold to mechanical stimuli. On day 25, the withdrawal threshold of the touched paw was significantly lower in rats treated with oxaliplatin (*n* = 10, 3.14 ± 0.89 g) than that in vehicle-treated rats (12.4 ± 2.94 g; *P* < 0.01). AC591 could significantly imped the alterations of the pain threshold evoked by oxaliplatin. After AC591 treatment, the withdrawal threshold to the non-noxious mechanical stimulus increased from 3.14 ± 0.89 g (oxaliplatin) to 9.75 ± 1.09 g (oxaliplatin + 20 g/kg AC591). Rats treated with AC591 in the absence of oxaliplatin did not show differences in the pain threshold as compared to the vehicle group (data not shown).

### AC591 Treatment Prevented Oxaliplatin-Induced Cold Hypoesthesia

We studied cold hypoesthesia in rats injected with 4 mg/kg of oxaliplatin on a cold plate set at 4 ± 0.2°C. By 4 weeks, oxaliplatin markedly reduced the latency to escape (*n* = 10, 128 ± 15 s) vs. the vehicle alone (204 ± 23 s; *P* < 0.001) or AC591 alone (221 ± 19 s; *P* < 0.001). AC591 at a dose of 20 g/kg markedly abrogated the alterations induced by oxaliplatin (197 ± 25 s with oxaliplatin+AC591 vs. 128 ± 15 s with oxaliplatin alone; *P* < 0.001) (**Figure 2C**). In the acetone test, no significant changes in withdrawal responses were observed in animals receiving the vehicle (**Figure 2D**). In contrast, the number of withdrawal responses significantly increased in oxaliplatin-treated rats when compared with the vehicle group on days 4, 7, 14, and 23 (*P* < 0.05). AC591 prevented the cold hypoesthesia and exerted a more pronounced effect (*P* < 0.01) at a dose of 20 g/kg. There was no statistically significant difference in the hot response thresholds between the groups of rats at any time point after oxaliplatin treatment, consistent with the clinical observations (**Figure 2E**).

### Neuropathological Analysis of DRG and Sciatic Nerve

**FIGURE 3:**
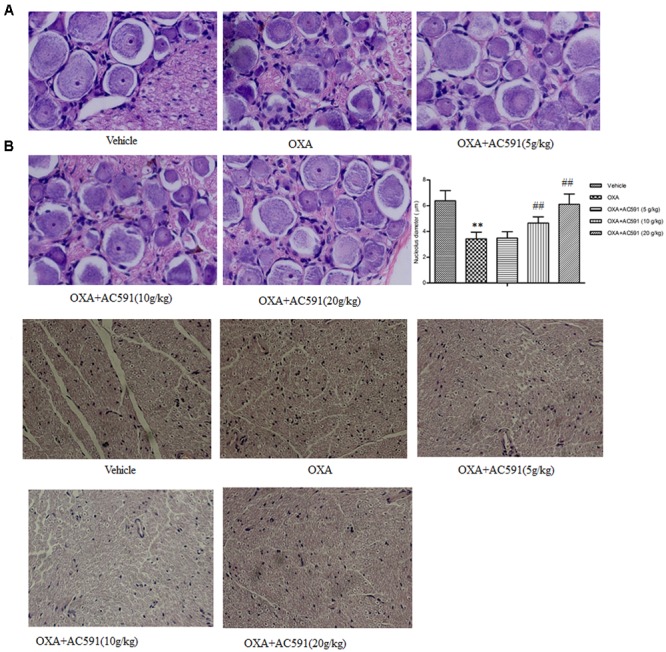
Effect of repeated administration of AC591 on histological change induced by oxaliplatin in rat DRG **(A)** and sciatic nerve **(B)**. Photographs for **(A)** were originally magnified 400, and for **(B)**, the photographs were magnified 100. Each value represents the mean of 5 rats per group. ^∗∗^*P* < 0.01 vs. vehicle; ^##^*P* < 0.01 vs. oxaliplatin.

The behavioral tests demonstrated that chronic oxaliplatin treatment induced sensitivity to mechanical and cold stimuli. Next, whether oxaliplatin exhibited neurotoxic changes in the structure of peripheral neurons was determined histologically. **Figures 3A,B** illustrated the histopathological changes on DRG and sciatic nerve specimens obtained after the final drug dosage. HE staining of lumbar spinal cord (L4–L5) DRG presented decreased nucleolar size and significant alteration in nuclear and cell body diameter in rats treated with oxaliplatin alone. Changes in DRG morphology were also previously documented in rats after treatment with multiple doses of oxaliplatin and other platinum drugs (Cavaletti et al., 2001). AC591 at doses of 10 and 20 g/kg significantly prevented the decrease in the nucleolar size of neurons highlighted in oxaliplatin-treated animals (**Figure 3A**). Examination of sciatic nerve sections by light microscopy did not exhibit remarkable histological abnormalities during oxaliplatin treatment, which may be explained by the fact that the main pathological changes in oxaliplatin-induced neuropathy occur predominantly in DRG but not sciatic nerves (**Figure 3B**) (De Grandis, 2007).

### Global Gene Expression in DRG after 4 Weeks of AC591 Treatment

**FIGURE 4:**
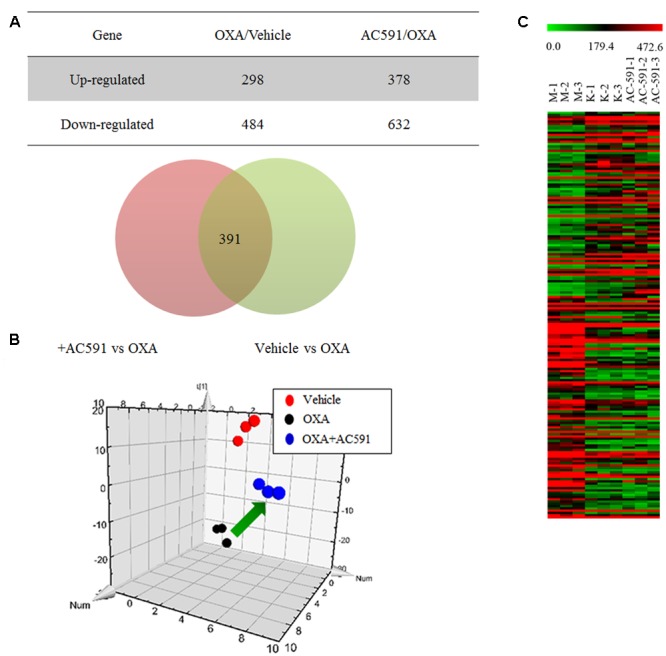
Transcriptome analyses on oxaliplatin-induced peripheral neuropathy and AC591-treated rats. **(A)** A Venn diagram illustrates there were 391 probe sets deregulated in oxaliplatin-induced peripheral neuropathy rats, but were rescued by the AC591 treatment. **(B)** A PCA plot using the 181 probe sets which were significantly reversed by the AC591. **(C)** A heat map shows the upregulation (in red) and downregulation (in green) patterns of the 181 probe sets. K: normal rats; M: PN rats treated with vehicle; AC591: PN rats treated with AC591 (20 g/kg).

We applied a microarray assay to detect 14,976 genes in total RNA samples of DRG specimens obtained from normal rats (*n* = 3), and PN rats treated with either AC591 (*n* = 3) or vehicle (*n* = 3). All samples passed quality control based on the inspection of the normalized intensity values, principle component analysis, and hybridization controls. Compared with normal rats, oxaliplatin-treated animals demonstrated ≥1.5-fold change in 782 genes and ≥2-fold change in 231 genes. Out of the 782 genes, 407 were annotated, and among them, 60 genes were related to immune or inflammatory response. In comparison with oxaliplatin, AC591 treatment corrected 391 (49.94%) out of 782 genes, and 181 genes among them were found to be significantly reversed (with >1.5-fold change in expression) (**Figure 4A**). A principle component analysis (PCA) plot using these 181 probe sets illustrated the variations in gene expression patterns between the various rat groups, and the AC591-treated ones were proximate to the vehicle controls (**Figure 4B**). A heat map showed 83 probe sets that were more abundant in the AC591-treated and vehicle control rats, while the other 98 oxaliplatin-induced probe sets were suppressed by AC591 (**Figure 4C**).

We next organized the AC591-affected genes into functional groups in order to obtain a better insight into the biological consequences of the gene expression changes. GO analysis using the DAVID program indicated that the highest proportion of the 391 genes were immune response (GO ID 6955) and defense response (GO ID 6952). As a result of AC591 administration, expression of a large number of genes related to wounding (17 genes, *P* = 6.8e^-3^) and inflammation (11 genes, *P* = 8.4e^-3^) was reduced to a level closer to that of the vehicle control. Other overrepresented GO pathways are shown in **Table 2**. KEGG pathway analysis was also used to discover the key signaling pathways and correlations between differentially expressed genes (Supplementary Table 2). Inflammation mediated by chemokine and cytokine signaling pathway (*P* = 0.0006) as well as Wnt signaling pathway (*P* = 0.0005) were identified as the most significant signaling mechanisms in enrichment analysis. The chemokine signaling pathway encompassed 13 differentially expressed genes: CCL4, CCL5, CCL6, CXCL10, CXCL11, CXCL13, CXCL16, CXCL9, HCK, NCF1, PIK3CD, STAT1, and VAV1.

**Table 2:** Functional module analysis for AC591-induced neuroprotection in oxaliplatin-induced peripheral neuropathy.

Term	Count	%	*P*-value
Immune response	23	6.3	1.2E-5
Inflammatory response	11	3.0	8.4E-3
Defense response	18	5.0	1.6E-3
Response to wounding	17	4.7	6.8E-3
Antigen processing and presentation	7	1.9	4.8E-3
Cell-substrate adhesion	7	1.9	2.6E-3
Cell-matrix adhesion	6	1.7	5.9E-3
Cytoskeleton organization	14	3.9	7.6E-3
Integrin-mediated signaling pathway	5	1.4	8.0E-3
Actin filament-based process	10	2.8	8.1E-3
Regulation of system process	13	3.6	1.8E-2
Regulation of T cell activation	7	1.9	2.0E-2
Response to DNA damage stimulus	11	3.0	2.5E-2
Protein complex biogenesis	15	4.1	2.6E-2
Blood circulation	8	2.2	2.8E-2
Smooth muscle cell proliferation	5	1.4	3.0E-2
regulation of leukocyte activation	8	2.2	3.4E-2
Cell cycle	14	3.9	9.7E-2
Neuron migration	4	1.1	9.7E-2
Response to mechanical stimulus	5	1.4	5.8E-2
Cell adhesion	15	4.1	5.7E-2
Biological adhesion	15	4.1	5.7E-2
Axon	4	1.1	7.2E-1
Response to oxidative stress	5	1.4	4.5E-1
Jak-STAT signaling pathway	6	1.7	1.2E-1
Neuron development	11	3	1.2E-1
Axonogenesis	5	1.4	4.8E-1

### AC591 Did Not Attenuate the Anti-tumor Activity of Oxaliplatin

**FIGURE 5:**
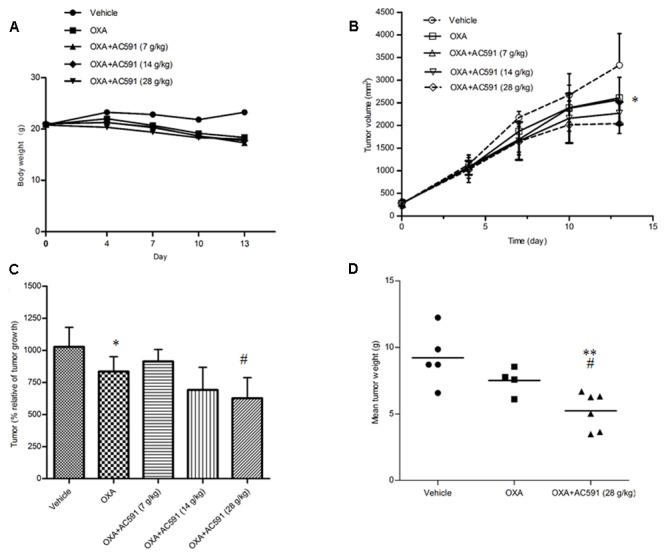
Effect of AC591 on the anti-tumor effect of oxaliplatin. **(A)** Effect of AC591 on body weight loss induced by oxaliplatin. **(B)** Effect of AC591 on tumor volumes. **(C)** Effect of AC591 on tumor growth. **(D)** Effect of AC591 on tumor weight. ^##^*p* < 0.05, compared with model group. ^∗^*p* < 0.05, ^∗∗^*p* < 0.01 compared with oxaliplatin treated group. Each value represents the mean of 8 mice per group. ^∗^*P* < 0.05, ^∗∗^*P* < 0.01 vs. vehicle; ^#^*P* < 0.05 vs. oxaliplatin.

In the present study, we also tested whether AC591 interfered with the tumor-reducing effects of oxaliplatin. Male BALB/c mice bearing CT26 tumors were randomly selected for treatment with saline, oxaliplatin (10 mg/kg/week, 2 weeks, i.p.) alone, and oxaliplatin+AC591 (7, 14, and 28 g/kg/d, i.g.). **Figure 5A** shows that oxaliplatin administration led to a substantial decrease in the body weight from day 6 after inoculation. Co-administration of AC591 exhibited no obvious effects on the weight loss induced by oxaliplatin. The mean tumor volume was similar in each of the five groups at the commencement of the experiment. On days 10 and 13, oxaliplatin significantly inhibited the increase of tumor volumes as compared to that of the vehicle in tumor cells implanted mice (*P* < 0.05, **Figures 5B,C**). AC591 did not attenuate, but rather resulted in the synergistic inhibition of the tumor growth as compared to oxaliplatin alone (*P* < 0.05, **Figures 5B,C**). After 2-week caliper measurement, the tumor was excised and weighed at 8.4 ± 2.1 g and 7.5 ± 1.0 g for vehicle control group and oxaliplatin-treated group, respectively. For oxaliplatin +AC591-treated group, tumor size averaged 5.2 ± 1.4 g, as significantly lower than that of vehicle (*P* < 0.001) or oxaliplatin-treated group (*P* < 0.05) (**Figure 5D**). These preliminary results suggested that AC591 did not attenuate, but rather enhance the anti-tumor activity of oxaliplatin.

### AC591 Attenuated the Oxaliplatin-Induced Neurotoxicity in Cancer Patients

#### Patient Characteristics

**FIGURE 6:**
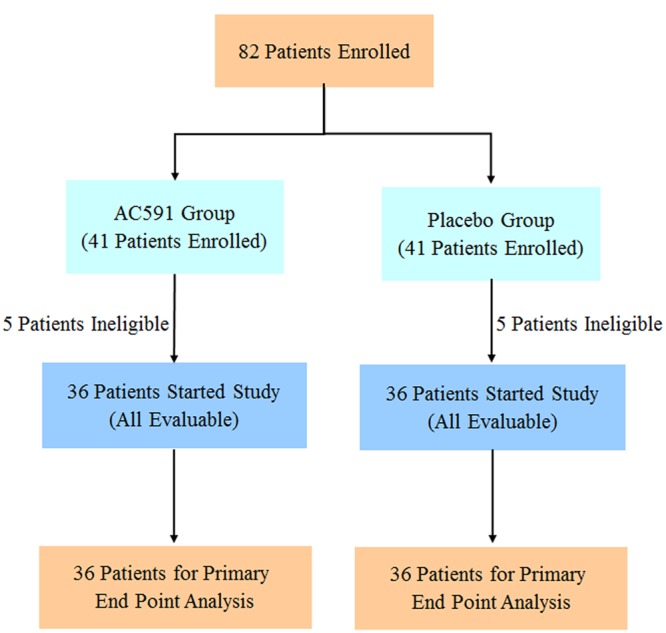
CONSORT statement flow diagram of patients included in the study.

For the clinical trial, 72 patients were recruited between July 1th, 2014 and October 31th, 2015, and randomly divided into the AC591-treated group and non-AC591 group (**Figure 6**). The baseline demographic and clinical characteristics are shown in **Table 3**. There was no significant difference between the two groups with respect to age, gender, performance status, the location of primary tumor, histological differentiation, and sites of distant metastasis. All the patients completed at least four treating cycles and were qualified for analysis. There were no drop-out cases.

**Table 3:** The basic characteristics of the study population.

Characteristics	AC591 group (*n*, %) (*n* = 36)	Non-AC591 group (*n*, %) (*n* = 36)	*P*-value
**Age (years)**			
≥50	34 (94.44)	35 (97.22)	N.S
<50	2 (5.56)	1 (2.78)	
Gender			
Male	22 (61.11)	20 (55.56)	N.S.
Female	14 (38.89)	16 (44.44)	
**Performance status**			
0	6 (16.67)	10 (27.78)	N.S.
1,2	30 (83.33)	26 (72.22)	
**Location of primary tumor**			
Colon	29 (91.67)	28 (87.5)	N.S.
Rectum	7 (8.34)	8 (12.5)	
**Histological differentiation**			
Well/moderately	16 (44.44)	16 (44.44)	N.S.
Poor/unknown	20 (55.56)	20 (55.56)	
**Sites of distant metastasis**			
Liver	12 (33.33)	11 (30.56)	N.S.
Lung	2 (5.56)	2 (5.56)	
Liver and lung	1 (2.78)	1 (2.78)	
Others	21 (58.33)	22 (61.10)	

#### Incidence of Oxaliplatin-Induced Peripheral Neurotoxicity

Data on the incidence of grade 0, 1, 2, 3, and 4 peripheral neuropathies are summarized in **Table 4**. After four cycles of drug treatment, 9 patients (25%) in the AC591 group exhibited grades 1–2 neurotoxicity, and no patients exhibited peripheral neuropathies with grades 3–4. In non-AC591 group, 20 patients (55.56%) experienced grades 1–2 neurotoxicity, and 3 patients (8.33%) were grades 3–4. The degree of neurotoxicity in AC591 group was lower than that of non-AC591 group (25% vs. 63.89%; *P* < 0.01).

**Table 4:** The occurrence of oxaliplatin-induced neurotoxicity and objective tumor response in the AC591 group and the placebo group.

Neurotoxicity	AC591 group (*n*, %) *n* = 36	Non-AC591 group (*n*, %) *n* = 36
**Occurrence of oxaliplatin-induced neurotoxicity**		
Grade 0	27 (75.00)	13 (36.11)
Grade 1	8 (22.22)	12 (33.33)
Grade 2	1 (2.78)	8 (22.22)
Grades 3–4	0	3 (8.33)
*Chi-square*	14.14	
*p*^a^	0.0027	
**Tumor response**		
CR	1 (2.78)	0
PR	6 (16.67)	5 (13.89)
SD	22 (61.11)	22 (61.11)
PG	7 (19.44)	9 (25.00)
*Chi-square*	1.341	
*p*^a^	0.7194	

*^a^chi-square test. CR, complete response; PR, partial response; SD, stable disease; PG, progression.*

#### Tumor Response

All patients completed four cycles of treatment, and the overall response rates (complete and partial response) were 19.45% in AC591 group and 13.89% in non-AC591 group. One patient in the AC591-treated group had a complete response. No significant differences in the tumor response rates were distinguished between the two groups (**Table 4**).

#### Adverse Events

AC591 used in this study appeared to be well-tolerated. Grades 3–4 adverse events related to AC591 were not displayed. The main adverse events in AC591 group were grade 1–2 nausea and vomiting, anorexia and constipation, which were caused by the chemotherapy (**Table 5**). With the exception of peripheral neuropathy, there was no significant difference in adverse events between the AC591 group and non-AC591 group (*P* > 0.05).

**Table 5:** Adverse events in the trial group and the control group.

Adverse events,*N* (%) (grades 1–2)	AC591 group (*n* = 36)	Placebo group (*n* = 36)
Asthenia/somnolence	0	0
Vomiting	3 (8.33)	2 (5.56)
Orthostatic hypotension	0	0
Nausea	9 (25)	7 (19.44)
Headache	0	0
Constipation	3 (8.33)	4 (11.11)
Dizziness	0	0
Anorexia	10 (27.78)	15 (41.67)
Insomnia	1 (2.78)	0
*Chi-square*	2.522	
*p*	0.6407	

## Discussion

Oxaliplatin or the oxaliplatin-based regimens are one of the first choices for colorectal cancer treatment. However, it is associated with the development of chronic painful neuropathy that is difficult to deal with. Although oxaliplatin-induced neuropathy has been studied for several years, the underlying mechanism is inconclusive and no effective therapy against this adversity is available. Our work proposed preventive effects of herbal medicine AC591 on oxaliplatin-induced neurotoxicity in animal model and cancer patients. We speculated that these findings may provide a beneficial choice for oxaliplatin neuropathy treatment.

In order to test whether AC591 is effective in attenuating oxaliplatin-induced peripheral neuropathy, we designed a neurotoxicity model using Wistar rats. Consistent with the previous reports, cold hyperesthesia and mechanical allodynia were established after 4 weeks of oxaliplatin injection (i.p., twice a week) (Ushio et al., 2012). The maximum level of cold hyperesthesia was observed at 4 weeks. Oxaliplatin-induced neuropathy is associated with damage in the peripheral sensory nerves (Argyriou et al., 2008), as is also clearly evident by the DRG changes in our work. Therefore, the rat model developed in this study was appropriate for mimicking human symptoms caused by oxaliplatin. All of the behavioral tests we conducted showed that AC591 effectively prevented the onset of mechanical allodynia and cold hyperalgesia in rats treated with oxaliplatin. Furthermore, morphological analyses revealed a decreased nucleolar size and significant alteration in nuclear and cell body diameter of DRG neurons in oxaliplatin-treated rats, which was abrogated by oxaliplatin’s association with AC591. This involvement of DRG neurons is a well-known characteristic of platinum-induced neurotoxicity, and our data are in agreement with the morphological changes observed by several groups (Cavaletti et al., 2001). Within the nervous system, oxaliplatin has been evidenced to accumulate preferentially in the DRG, and the extent to which oxaliplatin accumulates into DRG correlates with the degree of peripheral neuropathy (Screnci et al., 2000). Therefore, we hypothesized that AC591 may prevent the cellular uptake of oxaliplatin in DRG, and thus lead to the neuroprotective effect against oxaliplatin-induced neurotoxicity. In our future work, we would detect the concentration of oxaliplatin in DRG and confirm the effect of AC591 on uptake of oxaliplatin.

In order to gain deeper insights into the *in vivo* influences of AC591 on oxaliplatin-induced peripheral neuropathy, a genome-wide transcriptome analysis of DRG tissue was performed. PCA analysis illustrated that AC591 reversed oxaliplatin-induced peripheral neuropathy at a molecular level (181 genes significantly reversed) (**Figure 4**). According to the GO database, AC591 significantly enriched genes involved in immune and inflammatory responses (*P* = 1.2e^-5^ and 8.4e^-3^, respectively; **Table 2**). Genes involved in neuronal function, such as axonogenesis, neuron development, neuron migration, axon and blood circulation, were also induced in DRG of rats treated with AC591 (**Table 2**). Among AC591-downregulated genes, those responding to wounding or inflammation were reverted by AC591. Genes involved in response to mechanical stimulus (five genes, *P* = 5.8e^-2^) or regulation of leukocyte activation (eight genes, *P* = 0.034) were also inhibited by AC591. Signaling pathway analysis revealed that AC591 significantly suppressed the genes involved in inflammation mediated by chemokine and cytokine signaling pathway (Kasai et al., 2005). Previous studies have suggested that a myriad of cytokines and chemokines play an essential role in the development of peripheral neuropathy (Kiguchi et al., 2012). Cytokines such as IL-1β, IL-6, and TNF-α are upregulated in the injured peripheral nerves and elicit neuropathic pain (Moalem and Tracey, 2006; Thacker et al., 2007). These cytokines could activate other inflammatory cells, and desensitize the nociceptors in primary afferent neurons, e.g., TRPV1, a heat and chemical-sensitive cation channel (Binshtok et al., 2008; Kiguchi et al., 2010). The gene expression profile of DRG by microarray analysis showed that the neuroprotective action of AC591 may depend on modulation of multiple molecular targets and pathways involved in the downregulation of inflammation and immune response.

Given the findings obtained from rodent model, the preventive effect of AC591 on oxaliplatin-induced neurotoxicity was further confirmed in patients with colorectal tumor. After four cycles of chemotherapy, 9 patients (25.00%) in the AC591-treated group and 20 patients (55.55%) in the control group exhibited grades 1–2 sensory neuropathy. The difference of the incidence of neurotoxicity between the AC591 and control groups was significant (*P* < 0.01), indicating that AC591 was able to prevent the onset of oxaliplatin-induced neuropathy in cancer patients. AC591 execute the neuroprotective action without diminishing the antitumor efficacy of oxaliplatin. The tumor response rate was marginal with 19.45% in the AC591 group and 13.89% in the control group (*P* = 0.7194). Furthermore, AC591 appeared to be well tolerated during the trial without causing grades 3–4 adverse events. In animal experiment, AC591 gently improved the antitumor activity of oxaliplatin. However, AC591 did not show significant improvement in tumor response rate on patients. The differences in results may be explained by the species variations in pharmacokinetic behavior or pharmacodynamic activity, or both. Undoubtedly, there were some limitations in our study. There were only 72 patients participated in our trial, who underwent follow-up office visits every 2 weeks until 2 months after the completion of treatment. Hence, a large sample of patients should be enrolled in a future study, and a long term study is needed to further confirm our results.

## Conclusion

We demonstrated, for the first time, that herbal medicine AC591 ameliorated the oxaliplatin-induced neuropathy in the rat model and colorectal cancer patients, without affecting the anti-tumor activity of oxaliplatin. Based on our results, we contribute a plausible explanation and the opportunity for the evaluation of AC591, a standardized extract from traditional medicine formula HGWD, in the treatment or combination therapy of oxaliplatin–associated neuropathy. However, the exact mechanisms and active compounds responsible for the neuroprotective effects of AC591 are still unknown; thus, there is an urgent need to carry out further experiments to establish an empirical foundation for the application of AC591 as an adjunct therapy with oxaliplatin.

## Author Contributions

XCh and JH performed the majority of the experiments and wrote the manuscript. DW, XCa, XS, WL, YY, and CH supported several experiments. XW and PC designed the research and revised the manuscript.

## Conflict of Interest Statement

The authors declare that the research was conducted in the absence of any commercial or financial relationships that could be construed as a potential conflict of interest. The reviewer LX and handling Editor declared their shared affiliation, and the handling Editor states that the process nevertheless met the standards of a fair and objective review.
