# Supplementary material for: Herbal Medicine AC591 Prevents Oxaliplatin-Induced Peripheral Neuropathy in Animal Model and Cancer Patients
Source: Front Pharmacol. 2017 Jun 7;8:344. doi: 10.3389/fphar.2017.00344 (PMC5461429; doi:10.3389/fphar.2017.00344)
Supplement: Supplementary file 1 [file Table_1.DOC]

**Table 1**. Components of AC591 identiﬁed by LC-MS

| **No.** | **Peak** | **t_R (min)_** | **Compound** | **Molecular weight** | **Formula** | ***m/z* (ES+)** | ***m/z* (ES-)** | **Herb** |
| --- | --- | --- | --- | --- | --- | --- | --- | --- |
| 1 | P1 | 5.27 | Gallic acid | 170.1 | C_7_H_6_O_5_ | n | 169 | Paeoniae Radix Alba |
| 2 | P2 | 11.95 | Oxypaeoniflora | 496.5 | C_23_H_28_O_12_ | 519 | 495;555 | Paeoniae Radix Alba |
| 3 | P3 | 13.01 | Paeoniflorin sulfonate | 544.1 | C_23_H_28_0_13_S | n | 543 | Paeoniae Radix Alba |
| 4 | P4 | 18.37 | Albiflorin | 480.5 | C_23_H_28_O_11_ | 481;503 | 479;539 | Paeoniae Radix Alba |
| 5 | P5 | 20.10 | Paeoniflorin | 480.5 | C_23_H_28_O_11_ | 481;503 | 479;539 | Paeoniae Radix Alba |
| 6 | R1 | 24.35 | calycosin-7-O-β-D-glucoside | 446.4 | C_22_H2_2_O_10_ | 285;447 | 283 | Paeoniae Radix Alba |
| 7 | J1 | 25.12 | Rutin | 610.5 | C_27_H_30_O_16_ | 611 | 609 | Astragali Radix |
| 8 | P6 | 27.35 | Galloylpaeoniflorin | 632.6 | C_30_H_32_O_15_ | 655 | 631 | Jujubae Fructus |
| 9 | P7 | 27.77 | Benzoic acid | 122.1 | C_7_H_6_O_2_ | n | 121 | Paeoniae Radix Alba |
| 10 | P8 | 29.58 | Pentagalloylglucose | 940.7 | C_41_H_32_O_26_ | n | 939 | Paeoniae Radix Alba |
| 11 | C1 | 33.32 | Coumarin | 146.2 | C_9_H_6_O_2_ | 147 | n | Cinnamomi Ramulus |
| 12 | R2 | 41.90 | Ononin | 430.4 | C_22_H_22_O_9_ | 269;431 | n | Astragali Radix |
| 13 | R3 | 42.61 | Calycosin-7-O-β-D-glc-6''-O-acetate | 488.1 | C_24_H_24_O_11_ | 489;511;285 | n | Astragali Radix |
| 14 | R4 | 46.15 | 3,9-dimethoxypterocarpane-10-O-β-D-glucopyranoside | 462.0 | C_23_H_26_O_10_ | 485;301 | n | Astragali Radix |
| 15 | C2 | 46.60 | Cinnamic acid | 148.2 | C_9_H_8_O_2_ | n | n | Cinnamomi Ramulus |
| 16 | R5 | 47.47 | Calycosin | 284.3 | C_16_H_12_O_5_ | 285 | 283 | Astragali Radix |
| 17 | R6 | 48.47 | 2’-hydroxy-3’,4’-dimethoxyisoflavane-7-O-β-D-glucopyranoside. | 464.0 | C_23_H_28_O_10_ | 487;303 | n | Astragali Radix |
| 18 | C3 | 49.38 | Cinnamaldehyde | 132.2 | C_9_H_8_O | n | n | Cinnamomi Ramulus |
| 19 | R7 | 49.48 | Formononetin-7-O-β-D-glc-6''-O-malonate | 516.1 | C_25_H_24_O_12_ | 517;269 | n | Astragali Radix |
| 20 | C4 | 52.27 | unknown |  |  | 161;106 | n | Cinnamomi Ramulus |
| 21 | P9 | 54.18 | Benzoylalbiflorin | 584.6 | C_30_H_32_O_12_ | 607 | 583 | Paeoniae Radix Alba |
| 22 | P10 | 55.12 | Benzoylpaeoniflorin | 584.6 | C_30_H_32_O_12_ | 607 | 583 | Paeoniae Radix Alba |
| 23 | C5 | 57.66 | unknown |  |  | 163; |  | Cinnamomi Ramulus |
| 24 | R8 | 60.50 | Unknown |  |  | 353;295 | 329 | Astragali Radix |
| 25 | R9 | 61.33 | Formononetin | 268.3 | C_16_H_12_O_4_ | 269 | 267 | Astragali Radix |
| 26 | R10 | 62.67 | 3’-hydroxy-9,10-dimethoxypterocarpane | 300.3 | C_17_H_16_O_5_ | 301 | n | Astragali Radix |
| 27 | R11 | 63.09 | Astragaloside IV | 784.9 | C_41_H_68_O_14_ | 807 | 843 | Astragali Radix |
| 28 | R12 | 64.55 | 7,2-Dihydroxy-3,4-dimethoxyisoflavan | 302.3 | C_17_H_18_O_5_ | 303 | n | Astragali Radix |
| 29 | R13 | 67.59 | Astragaloside II | 827.0 | C_43_H_70_O_15_ | 849 | 886 | Astragali Radix |
| 30 | C5 | 66.30 | unknown |  |  | n | 295;233;147 | Cinnamomi Ramulus |
| 31 | Z1 | 69.67 | 6-gingerol | 294.4 | C_17_H_26_O_4_ | 317;137 | 293 | Zingiberis Rhizoma Recens |
| 32 | R14 | 70.20 | Isoastragaloside II | 827.0 | C_43_H_70_O_15_ | 849 | n | Astragali Radix |
| 33 | Z2 | 79.07 | 8-gingerol | 322.4 | C_19_H_30_O_4_ | 137 | 321 | Zingiberis Rhizoma Recens |
| 34 | Z3 | 80.48 | 6-shogaol | 276.4 | C_17_H_24_O_3_ | 277 | n | Zingiberis Rhizoma Recens |
| 35 | R15 | 80.63 | Acetylastragaloside I | 911.0 | C_47_H_74_O_17_ | 934 | n | Astragali Radix |
| 36 | Z4 | 85.33 | 10-gingerol | 350.5 | C_21_H_34_O_4_ | n | 349 | Zingiberis Rhizoma Recens |
