# Supplementary material for: Herbal Medicine AC591 Prevents Oxaliplatin-Induced Peripheral Neuropathy in Animal Model and Cancer Patients
Source: Front Pharmacol. 2017 Jun 7;8:344. doi: 10.3389/fphar.2017.00344 (PMC5461429; doi:10.3389/fphar.2017.00344)
Supplement: Supplementary file 2 [file Table_2.DOC]

**Table 2.** Top KEGG pathways enriched with differentially expressed genes reversed by AC591.

| **Pathway name** | **Number of genes** | **Genes** | **Fisher-Pvalue** |
| --- | --- | --- | --- |
| Inflammation mediated by chemokine and cytokine signaling pathway | 13 | CCL4, CCL5, CCL6, CXCL10, CXCL11, CXCL13, CXCL16, CXCL9, HCK, NCF1, PIK3CD, STAT1, VAV1 | 0.0006 |
| Wnt signaling pathway | 10 | TAX1BP3, TSC2, HBP1, SLC9A3R1, CSNK1G3, WIF1, WNT10a, WNT16, WNT2b, WNT3 | 0.0005 |
| Cytokine-cytokine receptor interaction | 15 | BMP7, CCL4, CCL5//CCL6, CD40, CSF1R, CXCL10, CXCL11, CXCL13, CXCL16, CXCL9, IL15, IL18, IL2RG, PDGFD | 0.0012 |
| Axon guidance | 6 | DPYSL5, EPHA4, NGEF, ROBO2, SEMA4B, SRGA | 0.0044 |
| PI3K-Akt signaling pathway | 13 | COL1A1, COL27A1, COL6A1, COL6A2, CSF1, FGF2, GNG8, IL4RA, IL7, LPAR3, PKN2, PRLR, THBS1 | 0.0101 |
| Regulation of actin cytoskeleton | 6 | ACTN3, ARHGEF12, MYLK, MYLPF, PPP1R12C, VCL, WASF2 | 0.0144 |
| Notch signaling pathway | 3 | APH1A, DTX3, RBPSUH | 0.0220 |
| Calcium signaling pathway | 6 | ATP2A1, MYLK, PHKA1, PRKACA, RYR1L, TNNC2 | 0.0247 |
| Toll-like receptor signaling pathway | 4 | MAP2K4, MAP3K7, PIK3R3, TOLLIP | 0.0281 |
| T cell receptor signaling pathway | 4 | LCK, MAP3K7, PIK3R3, VAV2 | 0.0428 |
